# β-Aminobutyric Acid Pretreatment Confers Salt Stress Tolerance in *Brassica napus* L. by Modulating Reactive Oxygen Species Metabolism and Methylglyoxal Detoxification

**DOI:** 10.3390/plants9020241

**Published:** 2020-02-13

**Authors:** Jubayer Al Mahmud, Mirza Hasanuzzaman, M. Iqbal R. Khan, Kamrun Nahar, Masayuki Fujita

**Affiliations:** 1 Department of Agroforestry and Environmental Science, Sher-e-Bangla Agricultural University, Sher-e-Bangla Nagar, Dhaka-1207, Bangladesh; jamahmud_bd@yahoo.com; 2 Department of Agronomy, Faculty of Agriculture, Sher-e-Bangla Agricultural University, Sher-e-Bangla Nagar, Dhaka-1207, Bangladesh; 3 Plant Systems Biology Laboratory, Department of Botany, Jamia Hamdard, New Delhi-110062, India; amu.iqbal@gmail.com; 4 Department of Agricultural Botany, Faculty of Agriculture, Sher-e-Bangla Agricultural University, Sher-e-Bangla Nagar, Dhaka-1207, Bangladesh; 5 Laboratory of Plant Stress Responses, Department of Applied Biological Science, Faculty of Agriculture, Kagawa University, Miki-cho, Kita-gun, Kagawa 761-0795, Japan

**Keywords:** abiotic stress, antioxidant defense, glyoxalase, ion homeostasis, organic acid, osmotic stress

## Abstract

Salinity is a serious environmental hazard which limits world agricultural production by adversely affecting plant physiology and biochemistry. Hence, increased tolerance against salt stress is very important. In this study, we explored the function of β-aminobutyric acid (BABA) in enhancing salt stress tolerance in rapeseed (*Brassica napus* L.). After pretreatment with BABA, seedlings were exposed to NaCl (100 and 150 mM) for 2 days. Salt stress increased Na content and decreased K content in shoot and root. It disrupted the antioxidant defense system by producing reactive oxygen species (ROS; H_2_O_2_ and O_2_^•−^), methylglyoxal (MG) content and causing oxidative stress. It also reduced the growth and photosynthetic pigments of seedlings but increased proline (Pro) content. However, BABA pretreatment in salt-stressed seedlings increased ascorbate (AsA) and glutathione (GSH) contents; GSH/GSSG ratio; and the activities of ascorbate peroxidase (APX), monodehydroascorbate reductase (MDHAR), dehydroascorbate reductase (DHAR), glutathione reductase (GR), glutathione peroxidase (GPX), superoxide dismutase (SOD), catalase (CAT), glyoxalase I (Gly I), and glyoxalase II (Gly II) as well as the growth and photosynthetic pigments of plants. In addition, compared to salt stress alone, BABA increased Pro content, reduced the H_2_O_2_, MDA and MG contents, and decreased Na content in root and increased K content in shoot and root of rapeseed seedlings. Our findings suggest that BABA plays a double role in rapeseed seedlings by reducing Na uptake and enhancing stress tolerance through upregulating the antioxidant defense and glyoxalase systems.

## 1. Introduction

Salinity is among the most detrimental stresses in plants, governing agricultural yield. When plants are exposed to excessive saline conditions, their metabolism gets debilitated, and growth and development are adversely affected. It has been estimated that salinity affects more than 20% of the cultivated land worldwide [1,2]. The occurrence of salt stress causes characteristic changes in plants from the time of occurrence until plant maturity [3]. Salt stress induces an initial water deficit, due to the relatively high solute concentrations in the soil, and also ion-specific stresses resulting from changes in K^+^/Na^+^ ratios [4], which lead to elevated levels of Na^+^ and Cl^-^ in the plants that hamper its growth and development [5]. It also impacts the physiological and molecular functioning of the photosynthetic components like chlorophyll, PSII and carotenoids, which are degraded, thereby decreasing the photosynthetic efficiency of the plants [4]. Reduced photosynthesis with increased salinity consequently may result in stomatal closure, a reduction in intracellular CO_2_ partial pressure and non-stomatal factors [6,7], and a reduction in protein concentration [8]. Reactive oxygen species (ROS) production is most prevalent during salinity stress and can damage cellular components such as protein, lipids and DNA, thus compromising vital cellular functions [9]. 

Maintenance of plant homeostasis is vital for the survival of plants during salt stress, which is achieved by ion uptake and compartmentalization for normal plant growth and development. To cope with the adverse effects of salinity, signaling molecules have been found to be effective [10]. Among these, the non-proteinogenic amino acid, β-amino butyric acid (BABA), is a xenobiotic compound which has evidently demonstrated its potentiality to induce resistance against abiotic and biotic stresses. β-aminobutyric acid-induced resistance through priming has been proposed as a way of generating crop varieties with an enhanced defensive capacity against abiotic stresses [11]. β-aminobutyric acid assists in enhancing plants’ resistance to abiotic stresses without compromising with the natural yield and productivity of the plant either through exogenous application [12] or through priming [13]. The potentiality of BABA in plant defense was further determined by the subjection of plants to salinity and submergence, where BABA levels were found to show simultaneous elevation in stressed plants [14]. An experimental demonstration of role of BABA in response to salt stress was suggested with *Arabidopsis* as the model organism. Expression patterns of marker genes are found in the SA pathway (PR-1, PR-5), the ABA pathway (RAB18, RD29A) [15]. It has also been made clear that the induction of salt stress tolerance in *Arabidopsis* is independent of functional SA signaling. Experimental evidences demonstrated that the priming of BABA induces ABA signaling, which is responsible for water stress tolerance in *Arabidopsis* [15]. Further roles of BABA in plants during abiotic stress conditions were illustrated in rice which was exposed to seed priming with BABA. Consequently, the priming of rice seedlings with BABA resulted in increased photosynthetic efficiency, increased mitochondrial activity and elevated levels of activity of nitrate reductase and antioxidant enzymes [16]. 

The priming of non-proteinogenic amino acids (BABA) is gaining attention for their efficient role in abiotic stress tolerance, with a cost-effective defense mechanism eliminating costly energy requirements. The exact mode of BABA priming and their molecular, physiological, and ecological aspects in plants are still under shadow and are expected to emerge as a great tolerance system for plants in the near future. Brassicaceae are important contributors to total oilseed production and most of them are classified as moderately salt tolerant, which would be more suitable for saline and dry lands in years to come. Therefore, it is very important to develop a strategy to improve the production of Brassicaceae plants under saline conditions. *Brassica napus* L. is an important and familiar member of Brassicaceae family. Therefore, in this study, we tried to evaluate the effect of BABA on Na accumulation, growth and biomass, water status, photosynthetic efficiency, cellular damage, and the performance of the antioxidant and glyoxalase systems of *B. napus* L. under NaCl stress during the early seedling stage. 

## 2. Results

### 2.1. Salt Accumulation and Na/K Ratio

Due to salt exposure, the Na content of *B. napus* L. in the shoot and root amplified in a concentration-dependent approach, and the roots confirmed elevated accumulation of the shoots (Table 1). Potassium content under the same condition showed the reverse result. In contrast to control plants, 100 and 150 mM NaCl stress decreased K content by 19% and 27% in shoot and 15% and 38% in root, respectively. Pretreatment of seedlings with BABA notably decreased only root Na content but decreased both shoot and root K content. Consequently, the supplementation of BABA before salt exposure lessened the ratio of Na and K by 15% and 23% in shoot and 18% and 36% in root under 100 and 150 mM NaCl stress, respectively, in contrast to their respective stress alone (Table 1).

### 2.2. Plant Growth and Biomass 

Salt toxicity harshly damages the growth and development of *B. napus* seedlings, including plant height, fresh weight (FW) and dry weight (DW) (Table 2). In contrast to control seedlings, plant height lessened by 9% and 13% due to 100 and 150 mM NaCl concentration, respectively. Seedlings pretreated with BABA considerably restored the plant height by 4% under 100 mM NaCl and 6% under 150 mM NaCl. A parallel tendency was obtained in cases of fresh and dry weight under the same treatment condition (Table 2).

### 2.3. Water Status and Osmotic Adjustment 

Salt stress negatively affected the water status of *B. napus* seedlings. A reduction in leaf relative water content (RWC) and a drastic enhancement of proline (Pro) content owing to salt stress established the water shortage situation of *B*. *napus*. In the present experiment, leaf RWC reduced by 6% and 11% in the seedlings under 100 and 150 mM NaCl stress, respectively, compared to control plants, whilst Pro level augmented by 109% and 184%, respectively (Table 2). In contrast, pretreatment of seedlings with BABA before salt exposure conspicuously improved the water status through adjusting the osmotic potential by enhancing leaf RWC and increasing the concentration of Pro in comparison with respective stress alone (Table 2).

### 2.4. Photosythetic Pigments

Salt stress hampered the photosynthesis process as it lessened levels of important photosynthetic pigments, including chl *a* and chl *b*. As a result, in comparison with control seedlings chl (*a*+*b*), content reduced by 44% due to 100 mM NaCl concentration and 64% due to 150 mM NaCl concentration. Pretreatment with BABA reinstates the chl (*a*+*b*) level by 60 and 91% under 100 and 150 mM NaCl concentration, respectively, in contrast to respective stress alone (Table 2).

### 2.5. Reactive Oxygen Species Generation, Oxidative Stress, and Membrane Damage

**Figure 1:**
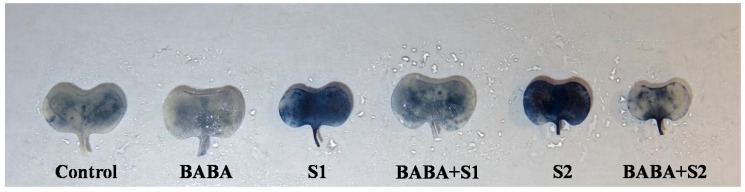
Effect of BABA on the superoxide (O_2_^•–^) accumulation of rapeseed (*Brassica napus* L.) seedlings exposed to different concentrations of salt stress. Here, BABA, S1 and S2 indicate 150 μM β-aminobutyric acid, 100 mM NaCl, and 150 mM NaCl, respectively.

**Figure 2:**
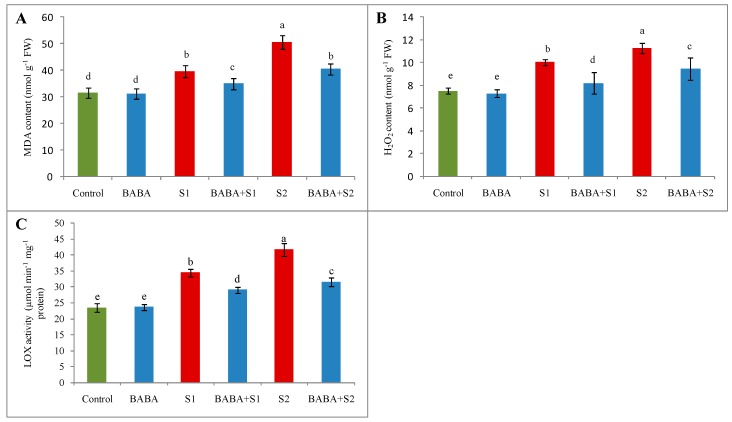
Effect of BABA on MDA content (**A**), H_2_O_2_ content (**B**), and LOX activity (**C**) of rapeseed (*Brassica napus* L.) seedlings exposed to different concentration of salt stress. Means (± SD) were calculated from three replications (n = 3) for each treatment. Average from three replications (n = 3) was used to determine mean (± SD) for each treatment. Letters with different values varied significantly at *p* ≤ 0.05 according to Fisher’s LSD test. Here, BABA, S1 and S2 indicate 150 μM β-aminobutyric acid, 100 mM NaCl and 150 mM NaCl, respectively.

In the present study, salt stress contributed to causing oxidative stress due to the overgeneration of ROS. Higher O_2_^•–^ was noticed in the leaves of *B*. *napus* by histochemical detection using nitroblue tetrazolium chloride (NBT). Salt stress affected leaves, showing significantly more deep blue spots/patches of O_2_^•–^ anions in comparison with control seedlings. However, pretreatment with BABA followed by salt stress reduced the spots/patches of O_2_^•–^ anions (Figure 1). Hydrogen peroxide (H_2_O_2_), another reactive free radical, enhanced by 33% and 50% under 100 and 150 mM NaCl stress, respectively, in contrast to control plants, whereas BABA pretreatment reduced H_2_O_2_ by 18% and 19% under 100 and 150 mM NaCl stress, respectively, in comparison with their respective stress treatment (Figure 2B). Moreover, LOX activity was enhanced by 46% under 100 mM NaCl and 77% under 150 mM NaCl, compared to control seedlings. Applying BABA reduced LOX activity by 16% and 24% under 100 and 150 mM NaCl stress, respectively, compared to their respective stresses (Figure 2C). Consequently, exposure of *B. napus* seedling to salt stress augmented malondialdehyde (MDA, a major index of lipid peroxidation) content by 26 and 60% at 100 and 150 mM naCl stress, respectively, but BABA pretreatment decreased MDA content notably under stress conditions (Figure 2A). 

### 2.6. Ascorbate–Glutathione Cycle

**Figure 3:**
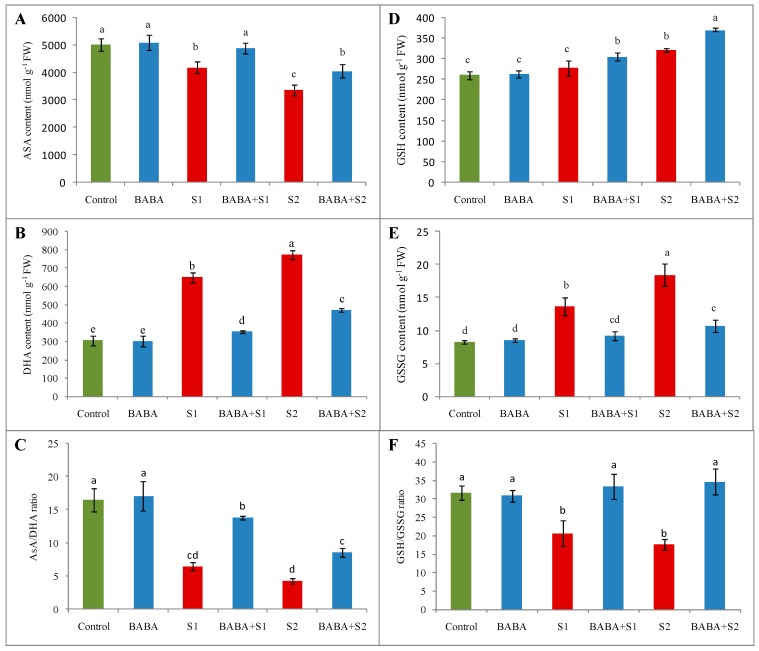
Effect of BABA on ascorbate (AsA) (**A**) and dehydroascorbate (DHA) (**B**) contents, AsA/DHA ratio (**C**), glutathione (GSH) (**D**) and oxidized GSH (GSSG) (**E**) contents, and GSH/GSSG ratio (**F**) of rapeseed (*Brassica napus* L.) seedlings exposed to different concentrations of salt stress. Average from three replications (n = 3) was used to determine mean (± SD) for each treatment. Letters with different values varied significantly at *p* ≤ 0.05 according to Fisher’s LSD test. Here, BABA, S1 and S2 indicate 150 μM β-aminobutyric acid, 100 mM NaCl and 150 mM NaCl, respectively.

Content of ascorbate (AsA) decreased by 17% in the *B. napus* seedlings under 100 mM NaCl stress and 33% under 150 mM NaCl stress. At the same time, dehydroascorbate (DHA) content augmented by 113% and 153% under 100 mM and 150 mM NaCl stress, respectively, in contrast to the control seedlings. Accordingly, the ratio between AsA and DHA notably reduced due to both concentrations of salt stress. However, in comparison with respective stress, BABA supplementation enhanced AsA content by 17% and 21%; decreased DHA content by 46% and 39%; and increased the AsA/DHA ratio by 115% and 98% under 100 and 150 mM NaCl concentrations, respectively (Figure 3A–C). 

Glutathione (GSH), the key non-enzymatic antioxidant, was enhanced by 7% due to 100 mM and 24% due to 150 mM NaCl stress, whilst oxidized GSH (GSSG) content enhanced by 65% due to 100 mM and 123% due to 150 mM NaCl stress. Consequently, the ratio between GSH and GSSG reduced by 35% and 44% under 100 and 150 mM NaCl concentrations, respectively. Conversely, in contrast to respective stress treatments, BABA supplementation enhanced GSH content further by 10% and 13%; reduced GSSG content by 32% and 42%; and enhanced the ratio of GSH and GSSG by 62% and 97% under 100 and 150 mM NaCl stress, respectively (Figure 3d–f).

**Figure 4:**
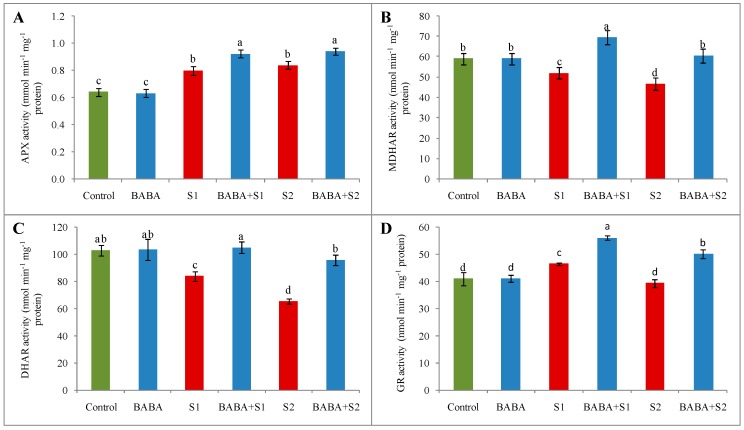
Effect of BABA on activity of APX (**A**), MDHAR (**B**), DHAR (**C**), and GR (**D**) of rapeseed (*Brassica napus* L.) seedlings exposed to different concentrations of salt stress. Means (± SD) were calculated from three replications (n = 3) for each treatment. Average of three replications (n = 3) was used to determine mean (± SD) for each treatment. Letters with different values varied significantly at p ≤ 0.05 according to Fisher’s LSD test. Here, BABA, S1 and S2 indicate 150 μM β-aminobutyric acid, 100 mM NaCl and 150 mM NaCl, respectively.

Ascorbate peroxidase (APX), monodehydroascorbate reductase (MDHAR), dehydroascorbate reductase (DHAR), and GSH reductase (GR) (four major enzymes of AsA-GSH pool) confirmed differentiable activity under salt stress condition (Figure 4). In contrast to control seedlings, function of APX enhanced by 25% and 31%; MDHAR activity reduced by 12% and 21%; and DHAR activity decreased by 18% and 36% under 100 and 150 mM NaCl stress, respectively (Figure 4A–C). However, the action of GR enhanced by 13% under 100 mM NaCl concentration but lessened by 4% under 150 mM NaCl concentration (Figure 4D). Compared to respective stress treatment, BABA supplementation before stress application enhanced the activity of all the antioxidant enzymes in the AsA-GSH pool (Figure 4). 

### 2.7. Activities of Superoxide Dismutase, Catalase, and Glutathione Peroxidase

**Figure 5:**
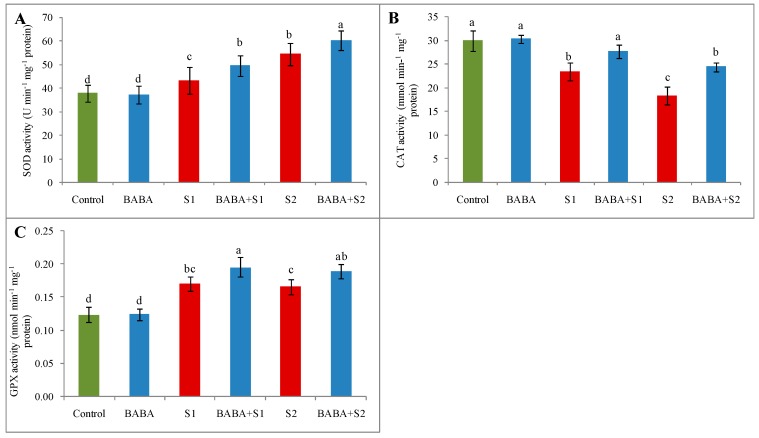
Effect of BABA on activity of superoxide dismutase (SOD) (**A**), catalase (CAT) (**B**), and glutathione peroxidase (GPX) (**C**) of rapeseed (*Brassica napus* L.) seedlings exposed to different concentrations of salt stress. Average from three replications (n = 3) was used to determine mean (± SD) for each treatment. Letters with different values varied significantly at *p* ≤ 0.05 according to Fisher’s LSD test. Here, BABA, S1 and S2 indicate 150 μM β-aminobutyric acid, 100 mM NaCl and 150 mM NaCl, respectively.

Compared to control treatment, superoxide dismutase (SOD) activity enhanced by 14% and 44% due to the exposure of seedlings to 100 and 150 mM NaCl concentrations, respectively. Applying BABA considerably increased SOD activity further (Figure 5A). Catalase activity lessened steadily with the increase in salt stress intensity. On the other hand, BABA-pretreated salt-affected seedlings reversed and restored the activity of catalase (CAT) by 18% and 33% under 100 and 150 mM NaCl stress, respectively (Figure 5B). In comparison with control seedlings, the action of GSH peroxidase (GPX) enhanced by 38% under 100 mM, and 35% under 150 mM, NaCl stress. Conversely, BABA pretreatment further improved the action of GPX in comparison with respective stress alone (Figure 5c).

### 2.8. Glyoxalase System and Methylglyoxal Content

**Figure 6:**
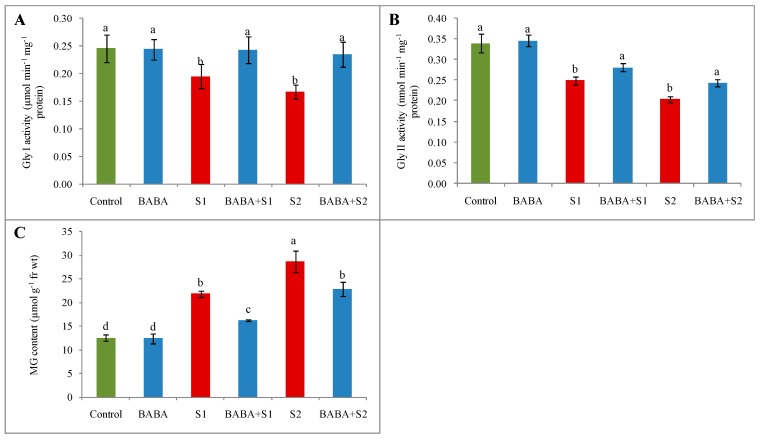
Effect of BABA on Gly I activity (**A**), Gly II activity (**B**) and MG content (**C**) of rapeseed (*Brassica napus* L.) seedlings exposed to different concentrations of salt stress. Average from three replications (n = 3) was used to determine mean (± SD) for each treatment. Letters with different values varied significantly at *p* ≤ 0.05 according to Fisher’s LSD test. Here, BABA, S1 and S2 indicate 150 μM β-aminobutyric acid, 100 mM NaCl and 150 mM NaCl, respectively.

Glyoxalase (Gly) I and Gly II activity decreased under salt stress. The activity of Gly I decreased by 20% and 32% and the activity of Gly II decreased by 27% and 40% under 100 and 150 mM NaCl stress, respectively. However, pretreatment with BABA followed by 100 and 150 MM NaCl concentration increased the activity of Gly I by 25% and 40% and the activity of Gly II by 13% and 19%, respectively, in comparison with their respective stresses (Figure 6A,B). Likely due to the action of BABA, the activity of both Gly I and Gly II was involved in scavenging toxic methylglyoxal (MG). Methylglyoxal increased by 74% and 129% under 100 and 150 mM NaCl stress, respectively. However, the enhanced efficiency of Gly I and Gly II due to BABA pretreatment was involved in the reduction in MG content by 24% and 20% under 100 and 150 mM NaCl stress, respectively (Figure 6c).

## 3. Discussion

Soil salinity has emerged as one of the most serious factors limiting plant growth and development and, ultimately, soil health [17]. Salt stress disturbs normal physiological, biochemical and molecular processes—most commonly manifested in stunted seed germination and seedling growth—that ultimately causes poor plant productivity and distinctly changed concentration of key biomolecules [18,19,20]. Salinity stress causes enormous alterations in the signaling pathways, as well as the regulation of gene expression, that affects the growth and development of plants [21]. In order to illustrate the impact of BABA-induced plant tolerance to salt stress, this study focused on physiological mechanisms to exhibit the reduction in plant sensitivity to stress upon BABA treatment. However, exogenous application of BABA can induce resistance against several biotic and abiotic stressors like salinity, drought, heat, cold and metal toxicity [22]. 

In the present study, salt treatment resulted in a higher Na^+^/K^+^ ratio in comparison to control. It has been shown that salinity-induced ROS formation can lead to programmed cell death (PCD), and a high cytosolic K^+^/Na^+^ ratio is essential for triggering salinity-induced PCD [23]. BABA, used against salt stress, decreased Na**^+^** and K**^+^** content and might be involved in salt tolerance. The reduction in the growth and dry matter of the salt-treated plants, as observed in this study, could be ascribed to the changes in plant water relation, resulting in a reduction in meristem activity as well as cell elongation [24], thereby inhibiting leaf expansion [25]. Similarly, the noticed decrease in the plant height and dry weight under salt stress might be due to Na^+^ and Cl^−^ ions accumulation in leaf tissues and could have resulted in growth hormone alteration, enzyme inactivation and stomatal closure and/or damage to photosynthetic machinery that, in turn, resulted in lower CO_2_ assimilation [26]. β-aminobutyric acid induced salt tolerance in *Arabidopsis* through enhanced ABA accumulation [15]. In the present study, BABA has shown to induce growth attributes under salinity and optimal conditions, suggesting that BABA promotes the accumulation of biomass. Such an enhancement of growth has also been observed in soybean, where plants are treated with BABA under a stressed and non-stressed environment [27]. Similar results were also observed when inducing stress tolerance upon salt stress [28].

Salinity enhances osmotic pressure, leading to a reduction in water absorbance. Cell division and differentiation are inhibited, which adversely affects metabolic and physiological processes [29]. In salt-stressed plant, there is a decrease in RWC, resulting in limited diffusion of H_2_O_2_ from its generation site [30]. The decreased RWC in rapeseed under increased salinity is indicative of a loss of cell turgor that leads to limited water availability for cell extension and expansion. Salinity obstructs the uptake and translocation of water due to the consequent high accumulation of Na^+^ and Cl^−^ ions in the cytoplasm, which leads to a nutrient imbalance [31]. It has been documented that Pro helps to mitigate stresses through different mechanisms, including the detoxification of toxic ions, protection of membrane integrity and stabilization of proteins/enzymes, and protects cell from ROS damage [32]. The proline content increased considerably, with an increase in salinity, but BABA application moderates this effect in studied plants. β-aminobutyric acid has been shown to induce proline accumulation in Arabidopsis and potato [33,34]. Misra and Misra [35] have reported that the up-regulation of proline biosynthesis enzymes (viz. pyrroline-5-carboxylate reductase and γ glutamyl kinase) and the down-regulation of proline oxidase activity led to an increased proline status, which helped to sustain the cell turgor in *Rauwolfia serpentina* under salinity stress.

In this study, photosynthetic pigments were decreased significantly in rapeseed due to salt stress. This also implies that a reduction in photosynthetic pigments occurred under stressed conditions and was significantly ameliorated on BABA application. β-aminobutyric acid priming might have a role in influencing the synthesis of photosynthetic pigments in the seedlings. Previously, Tabrizi et al. [36] have shown similar observation in maize seedlings. The decrease in chlorophyll content might have been due to a salt-induced increase in the activity of chlorophylase [37] and the instability of the pigment–protein complex, brought about by the elevated salinity level [38]. The decline in photosynthesis under salinity stress may be due to the inactivation of the PS II complex and a loss of chl pigments [39].

In the present study, the extent of oxidative stress was determined by the levels of superoxide, H_2_O_2_, and MDA content and LOX activity under 100 mM and 150 mM NaCl concentrations. However, in response to increasing salinity, many metabolic pathways are inhibited and proteins are catabolized. The increased ROS generation can alter normal cellular metabolism through increased oxygen-induced cellular damage, oxidative damage to DNA, proteins and membrane lipids, which led to the membrane lipid peroxidation [9]. Alternatively, BABA application reduced the level of ROS and H_2_O_2_ in salt-treated plants, which may also be a reason for reducing the lipid peroxidation of bio-membranes, as expressed by MDA content. β-aminobutyric acid treatment reduces the H_2_O_2_ accumulation that induces salt tolerance, and could be mediated by a faster osmoregulation process. The decline in the MDA content of seedlings raised from BABA application has been noticed in rice [40,41]. Furthermore, Wang et al. [42] stated that BABA decreased MDA content, polygalacturonase and pectinmethylesterase activities and enhanced cell wall polysaccharide content, resulting in the delayed senescence of cherry fruits. The emergence of this signal could induce the activation of antioxidant defense systems and ameliorate the oxidative damage initiated by ROS. Therefore, salt stress resistance may depend on the enhancement of the antioxidative defense systems. In this study, we demonstrated that BABA treatment is able to protect rapeseed against salt stress by augmenting the antioxidant defense system. Furthermore, BABA induced the up-regulation of antioxidant enzymes (SOD and CAT), which might play a role in removing the free radical which is correlated with increasing plant defense and reducing oxidative stress damage [28]. Moreover, numerous studies revealed that BABA is able to modulate the expression of defense-related genes involved in plant defense reactions. It was suggested that BABA induces a stress-induced morphogenic response in *Arabidopsis thaliana* that could be responsible for the BABA-induced resistance [43].

This suggests that BABA can induce the capacity to cope with salt stress in rapeseed plants. Other studies have shown that BABA induced greater accumulation of antioxidant defense components under salt stress [44,45,46], Cd stress [47] and drought stress [48]. The increase in APX and GR content in rapeseed, as well as the reduced AsA-GSH redox status accompanied by AsA–GSH cycle enzymes such as APX, MDHAR, DHAR and GR, clearly evidence that the AsA–GSH cycle plays a crucial role in scavenging ROS in rapeseed under salinity stress. It has been reported that APX and GR genes are up-regulated in plants, indicating less peroxisomal abundance of CAT activity [49]. Ascorbate peroxidase uses ascorbate as an electron donor in the first step of the ascorbate–glutathione cycle and is considered the most important plant peroxidase in H_2_O_2_ detoxification [50]. Previously, Abogadallahet al. [51] reported that APX-GR is the main H_2_O_2_ detoxifier that maintains ROS balancing in barnyard grass under salt stress. Recently, it has been shown that APX, CAT, GR and SOD antioxidant activities boosted protection against oxidative stress [52,53]. The elevated levels of GR activity could increase the GSH/GSSG ratio, which is required for ascorbate regeneration and the activation of several CO_2_ fixing enzymes in the chloroplasts [54], ensuring NADP^+^’s availability to accept electrons from the photosynthetic electron transport chain. The present study shows that MG production increased around 2–4-fold in rapeseed under salt stress; this might be due to a higher rate of glycolysis, amino acid and acetone metabolism, or other biochemical processes. β-aminobutyric acid treatment increased the Gly I and Gly II levels in salt treated rapeseed plants, which induces MG detoxification. However, the glyoxylase system composed of Gly I and Gly II regulates the enhanced production of MG under various environmental stresses including salinity [55,56], drought [20,57,58], toxic metals [59,60] and heat exposure [61,62]. Previously, Reddy and Sopory [63] observed that overexpression of the Gly I gene decreased the endogenous MG and enhanced the tolerance against MG and salt stress. Similarly, overexpression of the Gly II gene in rice enhances tolerance to MG and salt stress [56]. The glyoxylase system detoxifies MG in two-steps. In the first step, Gly I catalyzes the reaction of MG with GSH, and produces hemithioacetal, which is then converted to S-D-lactoylglutathione (SLG). In the second step, SLG is then hydrolysed to D-lactate, catalyzed by Gly II and, later, GSH-regenerated. At the end of the reaction, GSH is recycled because the availability of GSH is an important factor for detoxifying MG via the glyoxalase system [55].

## 4. Materials and Methods

### 4.1. Plant Materials, Experimental Conditions, and Treatments

Disease and pest free homogeneous rapeseed (*Brassica napus* L. cv. BARI Sharisha-13) seeds were used in this study. For sterilization, firstly seeds were dipped into 70% ethanol for five minutes and then rinsed with double distilled water thoroughly for several times. Nine centimeter Petri dishes containing six sheets of filter paper wetted by 10 mL distilled water were used to germinate seeds, maintaining equal distance. The Petri dishes containing seeds were transferred in a germinator for uniform and healthy germination (48 hours). Each Petri dish included 60 morphologically identical germinated seedlings. Then, the rapeseed seedlings contained in Petri dishes were shifted in a cultivation chamber (Iwaki; Asahi Techno Glass, Japan) under proper environment (light: 350 µmol photons m^−1^s^−2^; temperature: 25 ± 2 °C; relative humidity: 65%–70%). For plant nutrition, Hyponex solution (Hyponex, Japan; 5000-fold diluted) was added to the seedlings at regular intervals. Finally 6-d-old 50 plants of each Petri dish were pretreated with BABA, 150 μM for 48 h. Both BABA-pretreated and non-pretreated seedlings were then exposed to salt conditions (100 and 150 mM NaCl) and further grown for 48 h. A number of trials were performed before deciding the current doses of treatments. Leaves and roots of seedlings were collected to determine different important growth and physiological parameters as per standard techniques. The study was done following a completely randomized design (CRD) with six treatments (1. Control; 2. BABA; 3. 100 mM NaCl, S1; 4. BABA+S1; 5.150 mM NaCl, S2; 6. BABA+ S2) and repeated thrice. 

### 4.2. Measurement of Na^+^ and K^+^ Content 

Na**^+^** and K**^+^** content of shoots and roots of rapeseed seedlings were estimated by utilizing an atomic absorption spectrophotometer (Z-5000; Hitachi, Japan). A fresh sample of shoots and roots was collected and dehydrated in an oven for 72 h at 80 °C. Dry samples were then transferred for digestion separately into a strong acid mixture (HNO_3_:HClO_4_ = 5:1 v/v) at 80 °C for 48 h. Finally, spectrophotometer was used to read the absorbance of samples. A standard curve generated from a known concentration was used to estimate the shoots and roots Na**^+^** and K**^+^** content. 

### 4.3. Estimation of Plant Growth Parameters

Five plants were indiscriminately chosen from every treatment and used to determine the average height of plant in centimeters (cm). The fresh weight (FW) of a plant was measured from the average weight of 10 randomly selected fresh seedlings. The dry weight (DW) of plant was estimated after proper drying in the oven for 48 h at 80 °C. 

### 4.4. Determination of Leaf Relative Water Content

Whole fresh leaves were used to estimate leaf RWC, according to the the technique explained by Barrs and Weatherley [64]. The FW was recorded through weighing entire fresh leaves. The leaves were dipped in fresh water for 12 hours and weighed again after removal from water, to obtain the turgid weight (TW). Lastly, DW was estimated after dehydrating the turgid leaves for 48 h at 80 °C. The following formula was used to determine leaf RWC:

RWC (%) = [(FW−DW)/(TW−DW)] × 100(1)

### 4.5. Estimation of Proline Content

To determine the Pro level of rapeseed seedlings, 0.5 g leaves were blended well in 5 mL 3% sulfo-salicylic acid, keeping ice-cold conditions, by mortar and pestle, following the method explained by Bates et al. [65]. To get the supernatant, the extracted solution was centrifuged for 12 minutes at 11,500× *g*. Then, 1 mL supernatant was added with 1 mL acid ninhydrin and 1 mL glacial acetic acid, and the combination was incubated at 100 °C in a hot water bath. After 1 hour incubation, mixture solution was moved to clean the test tube and placed in an ice-containing box for proper cooling. After that, toluene (2 mL) was supplemented to the cooled solution and vortexed well. Finally, the absorbance of the chromophore-containing toluene was recorded spectrophotometrically at 520 nm wavelengths and the Pro level was estimated using a standard curve generated from the known concentration.

### 4.6. Measurement of Chlorophyll Content

To measure the content of chlorophyll (chl) *a* and chl *b* of rapeseed seedlings, 0.5 g fresh leaves were randomly selected and extracted in 80% (v/v) acetone (10 mL) according to the technique explained by Arnon [66]. Then, the extracted homogenate was centrifuged at 5000×*g* for 10 min. Finally, the absorbance of the solution was read after dilution. The 663 and 645 nm wavelength were used at UV-visible spectrophotometer for determining for chl *a* and chl *b* contents. 

### 4.7. Histochemical Detection of Superoxide Anion

Fresh leaves of rapeseed seedlings were dipped in 0.1% solution of NBT and incubated in a dark environment for 24 h, according to Nahar et al. [67]. After incubation, NBT treated leaves were blenched by hot/boiled ethanol and photographs were taken, where dark blue spots indicate the generation of O_2_^•−^. 

### 4.8. Estimation of Lipid Peroxidation 

Malondialdehyde content mostly determines the level of lipid peroxidation in the plant. The technique of Heath and Packer [68] was used to determine MDA, with few modifications, explained by Hasanuzzaman et al. [69]. Fresh leaves (0.5 g) were blended in 5% (w/v) trichloroacetic acid (TCA). Then, the extract was centrifuged at 11,500×*g* for 15 min to gain a clear supernatant, which (1 mL) was then added to 4 mL of thiobarbituric acid (TBA) reagent (solution of 0.5% of TBA and 20% TCA) and incubated at 95 °C in a hot water chamber. After 30 minutes, the mixture was transferred into a new clear test tube and placed in an ice-containing box for proper cooling. A UV-visible spectrophotometer was used to measure the absorbance at 532 nm and also at 600 nm (for correction of non-specific absorbance). At last, MDA level was estimated utilizing the extinction coefficient 155 mM^−1^ cm^−1^.

### 4.9. Determination of Hydrogen Peroxide Content

To determine H_2_O_2_ content, the protocol of Yu et al. [70] was used, in which fresh leaves (0.5 g) were blended in 3 mL of a potassium–phosphate (K–P) buffer with a concentration of 50 mM and pH 6.5. To obtain the clear supernatant, homogenates were then centrifuged for 15 min at 11,500×*g*. Two mL of supernatant was added to 666.4 µL mixture of 0.1% TiCl_4_ and 20% H_2_SO_4_ (v/v). Then the solution was incubated for 10 minutes, maintaining 25 °C temperature, and re-centrifuged again at 11,500× g for 12 min. The resulting final solution was read spectrophotometrically at 410 nm and the extinction coefficient 0.28 µM^−1^ cm^−1^ was used to calculate H_2_O_2_ level.

### 4.10. Extraction and Measurement of Ascorbate and Glutathione

A mixture of meta-phosphoric acid (5%) and EDTA (1 mM) was used as an extraction buffer to measure the components of AsA-GSH cycle. Leaves (0.5 g) were blended in 3 ml of extraction buffer and homogenized by ice-cooled mortar and pestle. Extracted homogenates were then centrifuged at 11,500×*g* for 15 min, maintaining a temperature of 4 °C. The content of AsA was measured according the procedures explained by Huang et al. [71] and Hasanuzzaman et al. [68]. Then, the clear supernatant was neutralized by 0.5 M K-P buffer (pH 7.0). For the reduction of the oxidized portion, 0.1 M of dithiothreitol was added to the neutralized solution. For measuring the content of total AsA and reduced AsA, changes in absorbance were recorded at UV-visible spectrophotometer (265 nm) after adding the final solution to a mixture of 100 mM K-P buffer (pH 7.0) and 1.0 U of ascorbate oxidase (AO). To estimate the oxidize portion of AsA (DHA content), reduced AsA was deducted from total AsA. For the determination of GSH-related components, enzymatic recycling was used according to Yu et al. [70], with slight amendments, as explained by Nahar et al. [69]. The very first supernatants (0.2 mL) were neutralized by 0.3 mL 0.5 M K-P buffer. After that, in the presence of GR, it was oxidized by 5,5-dithio-bis(2-nitrobenzoic acid) (DTNB). Finally the resulted solution was reduced by nicotinamide adenine dinucleotide phosphate (NADPH). Changes in absorbance were recorded spectrophotometrically at a 412 nm wavelength to measure GSH content. To measure the oxidized portion of GSH (GSSG), GSH was separated using 2-vinylpyridine derivatization. To measure the content of total GSH and GSSG, standard curves generated from known concentrations were used. To find out the reduced GSH content, GSSG was subtracted from total GSH. 

### 4.11. Enzyme Extraction and Assays

An ice-cold 50 mM K-P buffer (pH 7.0), 100 mM KCl, 1 mMAsA, 5 mM β-mercaptoethanol, and 10% (w/v) glycerol were mixed together to prepare the extraction buffer. Leaves (0.5 g) were blended in 1 mL of extraction buffer using pre-cooled mortar and pestle. Then, the resulted homogenates were centrifuged for 12 min at 11,500 *× g*. For executing the protein and enzyme assay procedure, the supernatant should be stored at 0–4 °C temperature.

A well-established protocol, described by Bradford [72], was followed to estimate protein content of plant samples, utilizing Bovine serum albumin as a protein standard.

The activity of Lipoxygenase (LOX, EC 1.13.11.12) was estimated according to the procedure explained by Doderer et al. [73]. The changes in sample absorbance were monitored at a 234 nm wavelength where, as a substrate, linoleic acid was used. The extinction coefficient 25 mM^−1^cm^−1^ was employed to calculate the activity of LOX.

To determine the action of the APX (EC: 1.11.1.11), the protocol explained by Nakano and Asada [74] was followed, where a reaction buffer was prepared using 50 mM K-P buffer (pH 7.0), 0.5 mMAsA, 0.1 mM H_2_O_2_, 0.1 mM EDTA. Then, the extracted solution was added to the reaction buffer and the absorbance was recorded at 290 nm. Finally, the action of APX was determined utilizing the extinction coefficient 2.8 mM^−1^ cm^−1^. The unit of APX activity was µmol min^−1^mg^−1^.

Hossain et al. [75] developed the method of determining the action of MDHAR (EC: 1.6.5.4) which was followed in this study. A solution buffer was arranged by mixing 50 mM Tris-HCl buffer (pH 7.5), 0.2 mM NADPH, 2.5 mM AsA and 0.5 units of AO (reaction initiator). Then, enzyme extract was added and the changes in absorbance were recorded at 340 nm for 60 s. The extinction coefficient 6.2 mM^−1^ cm^−1^was employed for the calculation of MDHAR. The unit of MDHAR activity was nmol min^−1^ mg^−1^.

Here, the methodology of Nakano and Asada [74] was used to quantify the action of DHAR (EC: 1.8.5.1). The reaction buffer was assayed to determine the activity of DHAR where a 50 mM K-P buffer (pH 7.0), 2.5 mM GSH, 0.1 mM EDTA, and 0.1 mM dehydroascorbic acid (DHA) was used. Finally, the extracted sample solution was added to the mixture of distilled water, buffer solution and DHA, and the changes in absorbance were recorded at 265 nm for 60 s. The extinction coefficient 14 mM^−1^ cm^−1^ was utilized for the estimation of DHAR. The action of DHAR was expressed as µmol min^−1^mg^−1^.

Hasanuzzaman et al. [69] developed the method of determining the action of GR (EC: 1.6.4.2) which was followed in this study. A reaction buffer was prepared by a 0.1 M K-P buffer (pH 7.0), 1 mM EDTA, 1 mM oxidized GSG, and 0.2 mM NADPH, at which point the enzyme extract was added and the reduction in absorbance was recorded at 340 nm for 60 s. The extinction coefficient 6.2 mM^−1^cm^−1^was utilized for the estimation of GR activity where the unit of GR activity was nmol min^−1^mg^−1^ protein.

A xanthine–xanthine oxidase system was utilized to estimate the activity of SOD (EC 1.15.1.1). The protocol described by El-Shabrawi et al. [76] was used to assay SOD. A 50 mM K-P buffer (pH 7.0), 2.24 mM NBT, xanthine oxidase (0.1 U), xanthine (2.36 mM) and catalase (0.1 U) were mixed together to prepare a reaction mixture for SOD. After that, an enzyme extract was added to the reaction mixture and the change in absorbance was recorded spectrophotometrically at a 560 nm wavelength. The unit of SOD activity was min^−1^ mg^−1^ protein.

To determine the action of CAT (EC: 1.11.1.6), the protocol developed by Hasanuzzaman et al. [69] was used. Following the procedure, a reaction mixer was developed utilizing a 50 mM K-P buffer (pH 7.0) and H_2_O_2_ (15 mM). After that, the extracted plant sample was added to the reaction mixer and the changes in absorbance were monitored at 240 nm for 60 s. The extinction coefficient 39.4 mM^−1^ cm^−1^ was employed for calculation. The unit of CAT activity was µmol min^−1^mg^−1^.

The activity of GPX (EC: 1.11.1.9) was estimated according to the protocol described by Elia et al. [77] after a minor change, as explained by Hasanuzzaman et al. [69]. A 100 mM K-P buffer (pH 7.0), 1 mM EDTA, 1 mM sodium azide (NaN_3_), 0.12 mM NADPH, 2 mM GSH, 1 U GR and 0.6 mM H_2_O_2_ (as a substrate) were mixed together to prepare the reaction mixture. Then, 20 mL of extracted plant sample was mixed with an assay buffer and the oxidation of NADPH was recorded at a 340 nm wavelength for 60 s. An extinction coefficient of 6.62 mM^−1^ cm^−1^ was utilized to calculate GPX activity and the unit of activity was nmol min^−1^mg^−1^ protein.

To measure Gly I (EC: 4.4.1.5) activity, a reaction buffer was prepared following the method explained by Hasanuzzaman et al. [69]. The reaction buffer contained 100 mM K-P buffer (pH 7.0), 15 mM MgSO_4_, 3.5 mM MG, 1.7 mM GSH and the extracted sample. Finally, the change in absorbance was recorded for 60 s at a 240 nm wavelength. The action of Gly I was estimated using the extinction coefficient 3.37 mM^−1^ cm^−1^. The unit of Gly I activity was μmol min^−1^mg^−1^ protein.

The protocol developed by Principato et al. [78] was used to determine the action of Gly II (EC: 3.1.2.6). An assay mixture including 100 mM Tris-HCl buffer (pH 7.2), 0.2 mM DTNB, and 1 mM *S*-D-lactoylglutathione (SLG) was mixed with the sample and the change in absorbance was read spectrophotometrically at 412 nm. An extinction coefficient 13.6 mM^−1^ cm^−1^ was employed to estimate action of Gly II, which was expressed as μmol min^−1^mg^−1^ protein. 

### 4.12. Determination of Methylglyoxal Content

To determine MG level, the protocol explained by Wild et al. [79] was used where leaves (0.25 g) were blended well in 2.5 mL 5% perchloric acid maintaining ice-cold conditions. Then, charcoal was used to decolorize the supernatant followed by neutralization with sodium carbonate. After that, a neutralizing solution was combined with sodium dihydrogen phosphate and N-acetyl-l-cysteine. Ten minutes later, the development of *N*-α-acetyl-S-(1-hydroxy-2-oxo-prop-1-yl) cysteine was documented by UV-visible spectrophotometer at 288 nm wavelengths. The level of MG was determined using a standard curve generated from known concentration. The unit of MG content was µmolg^−1^ FW. 

### 4.13. Statistical Analysis

The mean values of each parameter were calculated from three replications and subjected to analysis of variance (ANOVA) applying XLSTAT v. 2018 software [80]. The average of three replications (n = 3) was used to determine mean (± SD) for each treatment and mean differences were compared using Fisher’s LSD test where a variation of *p* ≤ 0.05 due to Fisher’s LSD test was considered significant.

## 5. Conclusions 

Our results suggest that salt stress damages rapeseed seedlings due to excessive accumulation of Na^+^ in a dose-dependent manner. It creates a severe oxidative stress through the overgeneration of ROS and enhances the production of MDA and MG, together with the increased activity of LOX. Moreover, it disrupted the antioxidant defense and glyoxalase system with the reduction in photosynthetic pigments and destroyed the water balance of seedlings. However, rapeseed seedling pretreated with BABA showed tolerance against salt toxicity as BABA up-regulated most of the antioxidants to scavenge toxic ROS and MG. We hope that the results of the study will help to elucidate the molecular and genetical basis of rapeseed seedlings under salt toxicity. 

## Figures and Tables

**Table 1:** Effect of β-aminobutyric acid (BABA) on Na content, K content, and the ratio of Na and K in both shoot and root of rapeseed (*Brassica napus* L.) seedlings exposed to different concentrations of salt stress. Average from three replications (n = 3) was used to determine mean (± SD) for each treatment and means of the same column were compared by letters, where letters with different values varied significantly at *p* ≤ 0.05 due to Fisher’s least standard difference (LSD) test.

Treatment	Shoot Na (µ mol g^−1^ DW)	Root Na (µ mol g^−1^ DW)	Shoot K (µ mol g^−1^ DW)	Root K (µ mol g^−1^ DW)	Shoot Na/K Ratio	Root Na/K Ratio
Control	11 ± 1.5 d	97 ± 13 d	118 ± 4.3 a	302 ± 10 a	0.10 ± 0.01 d	0.32 ± 0.05 d
BABA	11 ± 1.4 d	95 ± 12 d	115 ± 3.9 ab	301 ± 7 a	0.09 ± 0.01 d	0.31 ± 0.05 d
S1	990 ± 23 bc	1950 ± 94 b	96 ± 2.1 c	256 ± 4 c	10.29 ± 0.46 b	7.34 ± 0.30 bc
BABA+S1	977 ± 24 c	1727 ± 29 c	111 ± 3.1 b	294 ± 11 ab	8.79 ± 0.42 c	6.00 ± 0.34 c
S2	1062 ± 57 a	2272 ± 195 a	86 ± 1.1 d	185 ± 10 d	12.37 ± 0.71 a	12.29 ± 1.36 a
BABA+S2	1050 ± 53 ab	1941 ± 179 b	110 ± 3.3 b	252 ± 40 c	9.57 ± 0.71 bc	7.87 ± 1.70 b

Here, BABA, S1 and S2 indicate 150 μM β-aminobutyric acid, 100 mM NaCl and 150 mM NaCl, respectively.

**Table 2:** Effect of BABA on plant height, fresh weight (FW), dry weight (DW), leaf relative water content (RWC), Pro content, chl *a*, chl *b* and chl (*a*+*b*) content of rapeseed (*Brassica napus* L.) seedlings exposed to different concentrations of salt stress. Average from three replications (n = 3) was used to determine mean (± SD) for each treatment, and means of the same column were compared by letters where letters with different values varied significantly at *p* ≤ 0.05 due to Fisher’s LSD test.

Treatment	Plant Height (cm)	Fresh Weight, FW (mg Seedling^−1^)	Dry Weight, DW (mg Seedling^−1^)	Leaf Relative Water Content, RWC (%)	Proline, Pro Content (μmolg^−1^ FW)	Chl *a* (mg g^−1^ FW)	Chl *b* (mg g^−1^ FW)	Chl (*a*+*b*) (mg g^−1^ FW)
Control	5.28 ± 0.14 a	69.2 ± 1.9 ab	7.98 ± 0.26 a	91.6 ± 0.9 a	1.33 ± 0.05 e	0.68 ± 0.03 a	0.28 ± 0.01 a	0.95 ± 0.016 a
BABA	5.31 ± 0.07 a	70.5 ± 1.5 a	8.16 ± 0.24 a	91.3 ± 0.6 a	1.34 ± 0.06 e	0.67 ± 0.03 a	0.27 ± 0.01 a	0.95 ± 0.022 a
S1	4.83 ± 0.06 c	64.1 ± 2.3 c	7.17 ± 0.26 cd	86.2 ± 0.8 c	2.78 ± 0.08 d	0.42 ± 0.01 d	0.11 ± 0.004 d	0.53 ± 0.004 d
BABA+S1	5.04 ± 0.06 b	68.1 ± 2.0 ab	7.78 ± 0.23 ab	88.7 ± 0.5 b	3.46 ± 0.13 c	0.59 ± 0.01b	0.27 ± 0.005 b	0.85 ± 0.008 b
S2	4.60 ± 0.08 d	60.0 ± 1.8 d	6.78 ± 0.47 d	81.4 ± 0.9 d	3.78 ± 0.11 b	0.26 ± 0.02 e	0.08 ± 0.008 e	0.34 ± 0.013 e
BABA+S2	4.88 ± 0.06 c	66.0 ± 2.1 bc	7.41 ± 0.25 bc	86.2 ± 0.4 c	4.93 ± 0.19 a	0.47 ± 0.02 c	0.18 ± 0.01 c	0.65 ± 0.013 c

Here, BABA, S1 and S2 indicate 150 μM β-aminobutyric acid, 100 mM NaCl and 150 mM NaCl, respectively.
